# Guanylate cyclase-C Signaling Axis as a theragnostic target in colorectal cancer: a systematic review of literature

**DOI:** 10.3389/fonc.2023.1277265

**Published:** 2023-10-20

**Authors:** Moein Piroozkhah, Ali Aghajani, Pooya Jalali, Arvin Shahmoradi, Mobin Piroozkhah, Younes Tadlili, Zahra Salehi

**Affiliations:** ^1^ Basic and Molecular Epidemiology of Gastrointestinal Disorders Research Centre, Research Institute for Gastroenterology and Liver Diseases, Shahid Beheshti University of Medical Sciences, Tehran, Iran; ^2^ Department of Laboratory Medicine, Faculty of Paramedical, Kurdistan University of Medical Sciences, Sanandaj, Iran; ^3^ School of Medicine, Tehran University of Medical Sciences, Tehran, Iran; ^4^ Department of Molecular Cell Biology, Microbiology Trend, Faculty of Basic Sciences, Islamic Azad University, Central Tehran Branch, Tehran, Iran; ^5^ Hematology-Oncology and Stem Cell Transplantation Research Center, Tehran University of Medical Sciences, Tehran, Iran

**Keywords:** Guanylate cyclase-C Signaling Axis, Guanylyl cyclase C, guanylin, uroguanylin, colorectal cancer, therapeutic target

## Abstract

**Introduction:**

Colorectal cancer (CRC) is a devastating disease that affects millions of people worldwide. Recent research has highlighted the crucial role of the guanylate cyclase-C (GC-C) signaling axis in CRC, from the early stages of tumorigenesis to disease progression. GC-C is activated by endogenous peptides guanylin (GU) and uroguanylin (UG), which are critical in maintaining intestinal fluid homeostasis. However, it has been found that these peptides may also contribute to the development of CRC. This systematic review focuses on the latest research on the GC-C signaling axis in CRC.

**Methods:**

According to the aim of the study, a systematic literature search was conducted on Medline and PubMed databases. Ultimately, a total of 40 articles were gathered for the systematic review.

**Results:**

Our systematic literature search revealed that alterations in GC-C signaling compartments in CRC tissue have demonstrated potential as diagnostic, prognostic, and therapeutic markers. This research highlights a potential treatment for CRC by targeting the GC-C signaling axis. Promising results from recent studies have explored the use of this signaling axis to develop new vaccines and chimeric antigen receptors that may be used in future clinical trials.

**Conclusion:**

The findings presented in this review provide compelling evidence that targeting the GC-C signaling axis may be an advantageous approach for treating CRC.

## Highlights

- 1. The progression of colorectal cancer (CRC) is attributed to GC-C, GN, and UG peptides.
- 2. In CRC, the GN and GU expression is lost due to abnormal APC-β-catenin-TCF transcriptional regulation in the early phase of tumor formation. This impairs the GC-C signaling axis and disrupts intestinal homeostasis, contributing to tumor initiation.
- 3. When the GC-C axis is blocked, there is excessive cell growth, increased crypt size, and reduced cell differentiation in the secretory lineage.
- 4. Targeting GC-C with CAR-T cells could be an innovative and practical approach to treating advanced stages of CRC or those resistant to traditional therapies.
- 5. The repurposing of GC-C signaling axis-targeted treatments developed for other diseases is a promising strategy for CRC treatment. Further research is needed to investigate their safety and efficacy.

## 1 Introduction

Globally, colorectal cancer (CRC) is the third most prevalent form of cancer and has the second-highest mortality rate among all cancers. The CRC mortality rate has been decreasing at a rate of around 2% each year during the last decade. Projections indicate that by the end of 2023, there will have been over 150,000 new cases and about 53,000 fatalities (1). Population-wide shifts toward better lifestyles (e.g., less consumption of red and processed meat) and increased participation in the cancer screening program have been attributed to a decline in colorectal cancer incidence (2, 3). However, this improvement has been made since the early 2000s, primarily because of increased colonoscopy screening methods and the removal of precursor lesions. The primary prevention of colorectal cancer continues to be the most efficient way of reducing the rising global burden of this disease (4–7).

Symptoms of CRC in its early stages are unspecific, which might lead to missed diagnoses or mistaken assessments. Therefore, most cases of CRC are detected in the late stages (8). Surgical resection, radiation therapy before surgery (for rectal cancer), chemotherapy after surgery (for stages III/IV and high-risk stage II colon cancer), and targeted therapy are the most common therapies for CRC (9, 10). Although development in anticancer therapy, particularly immune checkpoint inhibitors (ICIs), has revolutionized CRC treatment, only a fraction of patients respond to these treatments appropriately, and the reason for failure in other patients has not yet been known properly (7, 11). Thus, understanding the molecular mechanism in cancers is crucial for creating reliable diagnostic and prognostic biomarkers in both practice and research (12). Significant advancements in microarray and high-efficiency sequencing technologies like next-generation sequencing have accelerated the exploration and identification of the essential genetic or epigenetic modifications in carcinogenesis, tumor growth, disease recurrence, and metastasis in CRC as well as the discovery of cancer biomarkers with the potential for the development of novel diagnostic, prognostic, and therapeutic techniques (13, 14). Over the past few decades, key driver and passenger genes in CRC have been recognized (15). Auspiciously, apart from the main oncogenes leading to CRC, numerous other genes implicated in CRC development and progression are being discovered and may be exploited as new biomarkers in clinical settings to predict prognosis or therapy response in the future (16).

The transmembrane receptor GC-C is selectively expressed from duodenal to rectal intestinal epithelial cells (17). GC-C is activated by endogenous peptides GN and UG, as well as exogenous ligands such as heat-stable enterotoxins (STs), which are secreted by diarrhea-producing enterotoxigenic E. coli (ETEC) (18).

**Figure 1:**
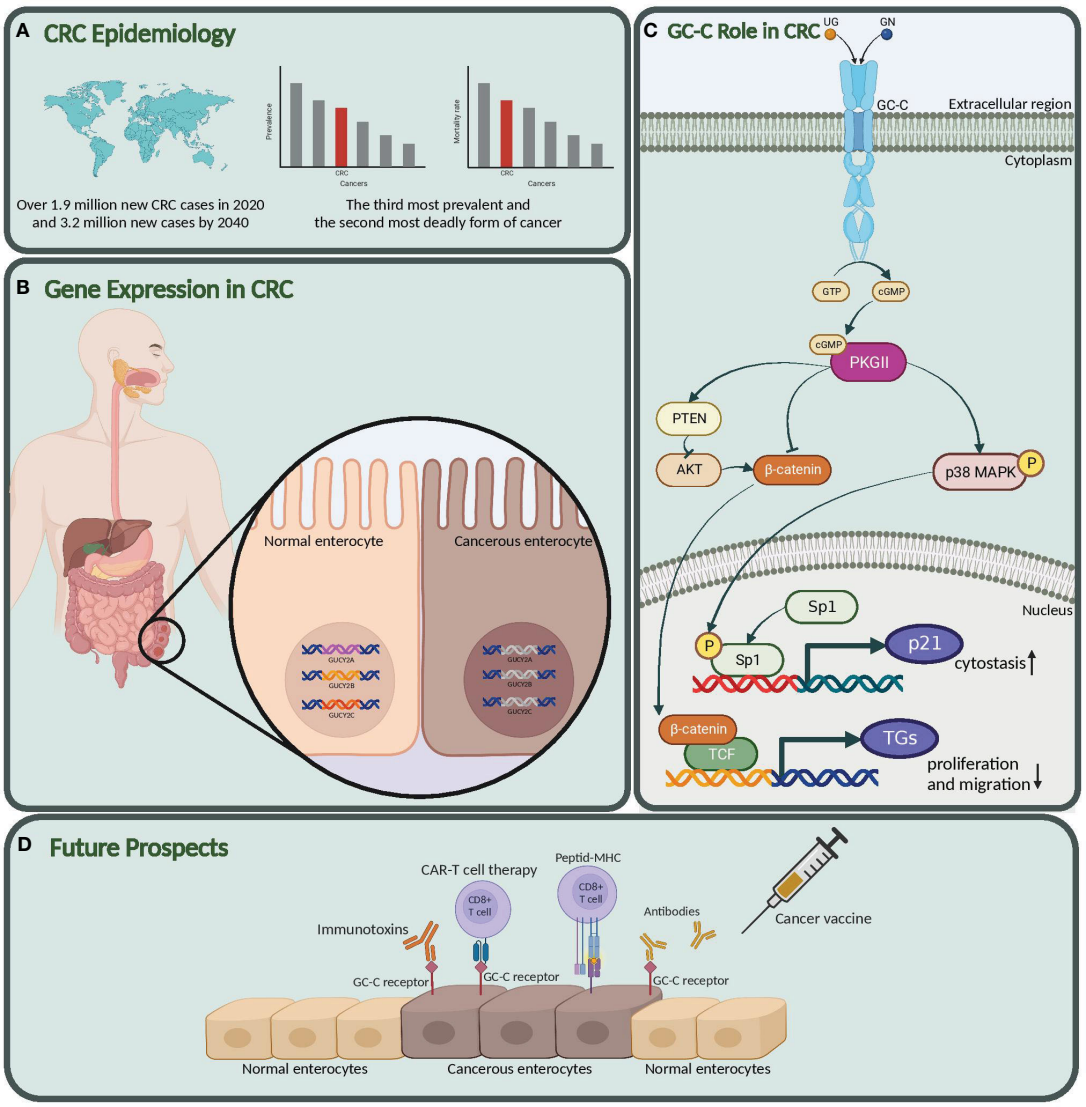
Graphical abstract. **(A)** CRC epidemiology. Global impact of colorectal cancer. **(B)** Gene expression in CRC. suppressed expression in *GUCY2C*, *GUCA2A*, and *GUCA2B* in Cancerous vs. Normal Enterocytes. **(C)** GC-C role in CRC. Here, we also explored the molecular mechanism and role of this pathway in the pathogenesis of colon cancer. **(D)** Future prospects. Our study highlights the potential of the Guanylate cyclase-c (GC-C) signaling pathway as a diagnostic biomarker, prognostic indicator, and target for new therapeutic strategies in colorectal cancer. *GUCY2C*, Guanylate Cyclase 2C; *GUCA2A*, Guanylyl cyclase-activating protein 2A; *GUCA2B*, Guanylate Cyclase Activator 2B; GC-C, Guanylate Cyclase-C; CRC, Colorectal Cancer. Created with BioRender.com.

GN and UG are very close in structure and biological functions. The GC-C signal transduction controls the equilibrium of liquid and electrolyte transport and secretion in the digestive tract (19, 20). However, new findings indicate that these novel peptides have diverse physiological roles alongside those previously documented for the control of homeostasis and might contribute to the tumorigenesis of colorectal adenocarcinoma (21–25). The coincidence of the emergence and development of CRC with the presence of GC-C, GN, and UG proteins, along with their encoding genes *GUCY2C*, *GUCA2A*, and *GUCA2B*, respectively, has piqued the interest of researchers. Particularly, GUCY2C has been found to play a regulatory role in intestinal inflammation and inflammatory bowel disease pathology. Studies of intestinal inflammation in Gucy2c knockout mice have reported the impairment of the epithelial barrier, increased invasiveness of pathogenic bacteria, and alterations in the intestinal microbiota (26, 27). Overall, these data suggest that IBD susceptibility may be mediated by alterations in the intestinal microbiota caused by changes in intestinal ion transportation regulated by GC-C. As a result, we have undertaken a comprehensive review of the literature to explore the relationship between these genes and CRC. Our systematic review not only examines how these genes contribute to CRC development but also emphasizes their potential uses in diagnosing and treating the disease, illustrated as schematic abstract (**Figure 1**).

## 2 Materials and methods

### 2.1 Search strategy and data extraction

This study presents a systematic review focusing on the role of the Guanylate cyclase-C Signaling Axis CRC. The search for relevant articles was conducted until May 27, 2023, extensively utilizing the Medline and PubMed databases. The search algorithm incorporated key terms related to the Guanylate cyclase-C Signaling Axis, including Guanylyl cyclase C, Guanylin, uroguanylin, their associated genes, and colorectal cancer. Meta-analyses, reviews, case reports, correspondences, and personal opinions studies were excluded (**Table 1**).

**Table 1:** Search strategy performed in PubMed.

Query	Search Algorithm	Number of Records
#1	(((GUCA2B[Title/Abstract]) OR (GUCY2C[Title/Abstract]) OR (GUCA2A[Title/Abstract]) OR (“guanylyl cyclase C”[Title/Abstract]) OR (“Guanylate cyclase”[Title/Abstract]) OR (“Gc-c receptor”[Title/Abstract]) OR (“guanylate”[Title/Abstract]) OR (“Guanylin”[Title/Abstract]) OR (“uroguanylin”[Title/Abstract])) AND ((Colorectal[Title/Abstract]) OR (intesti*[Title/Abstract]) OR (gastro*[Title/Abstract]) OR (colon[Title/Abstract])) AND ((cancer[Title/Abstract]) OR (malignan*[Title/Abstract]) OR (carcino*[Title/Abstract]) OR (neoplasm*[Title/Abstract])))	69

### 2.2 Inclusion and exclusion criteria, population, intervention, and outcomes

During the initial screening process, duplicate articles and those published before 2018 were excluded. Additionally, only articles written in English were included to mitigate potential language and publication biases. Two experts (MEP and AA) independently evaluated article content, demographics, research methodologies, and outcomes to identify potentially eligible studies. Inclusion criteria encompassed studies involving human, animal, *in vivo*, and *in vitro* colorectal cancer samples, focusing on applying the guanylate cyclase-c signaling axis for diagnostic, prognostic, therapeutic targeting, and survival assessment purposes. Studieswith inadequate research designs or misaligned outcomes were excluded from consideration. The search terms and methodologies applied (including database searches, screening, selection, and inclusion criteria) were consistently employed to ensure comprehensive coverage of relevant articles, adhering to the PRISMA statement of 2020 (28).

### 2.3 Risk of bias

Two assessors (MEP and AA) conducted independent assessments to evaluate potential bias. In cases of disagreement, a third assessor (PJ) was consulted. The evaluation of bias employed the Risk Of Bias In Non-randomized Studies - of Interventions (ROBINS-I) tool, which encompassed eight domains: bias arising from confounding; bias in participant selection; bias in classification; bias due to deviations in intended interventions; bias resulting from missing data; bias due to outcome measurement; bias in the selection of reported results; and overall bias. This rigorous approach ensured a comprehensive assessment of potential biases in the included studies (29).

## 3 Results

**Figure 2:**
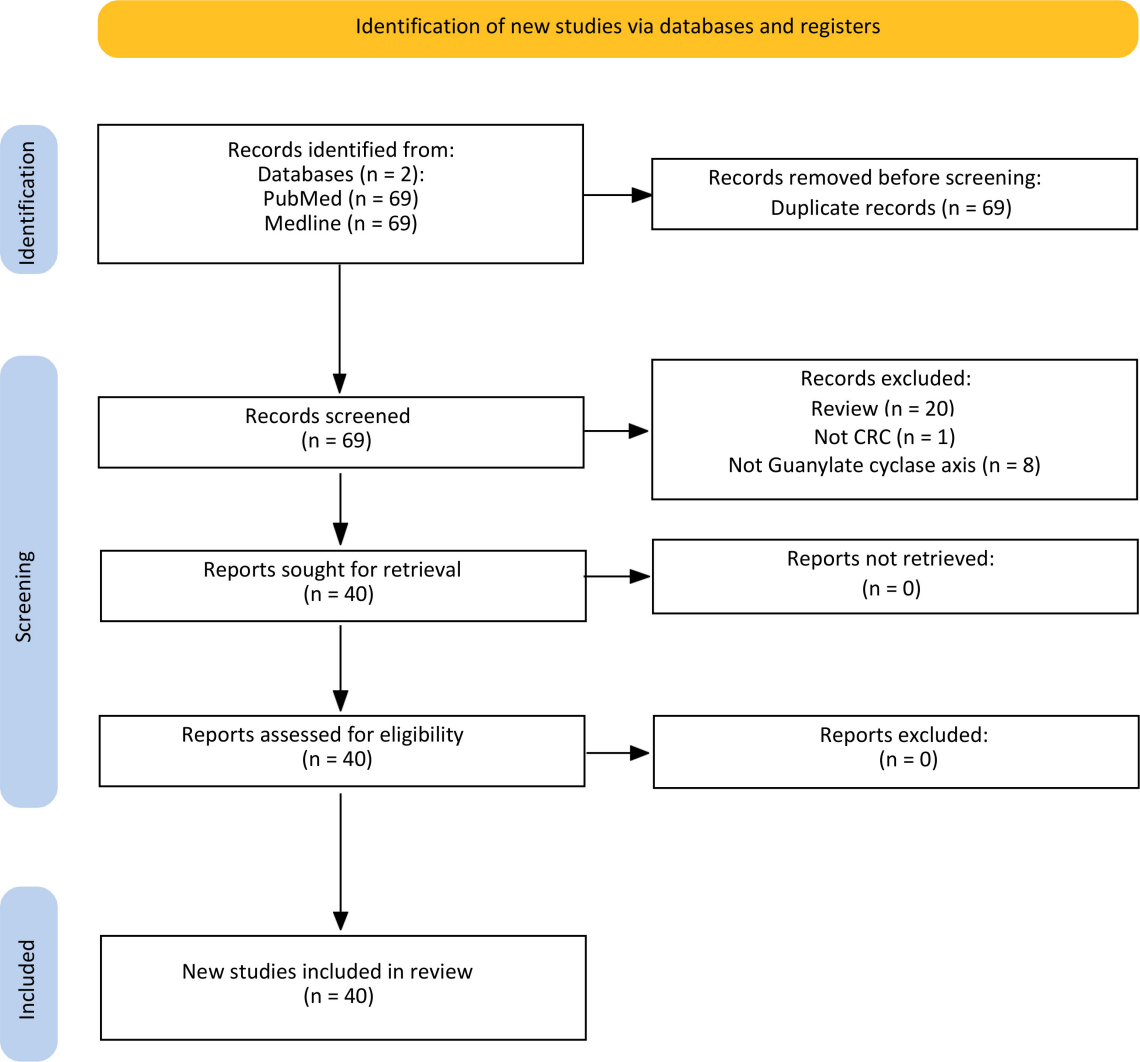
Flow chart of the literature search for Guanylate cyclase-C signaling pathway related to colorectal cancer.

**Figure 2** is a flowchart of the searching, screening, and process of the references we selected from the literature. According to the search strategy, 138 published papers were found in each database. After omitting 69 papers as duplicate articles, two separate team members screened 69 titles and abstracts. Twenty-nine articles were eliminated after the title and abstract screening (Review n=20) (Not CRC n=1) (Not Guanylate cyclase axis n=8), and the remaining 40 articles were evaluated for eligibility. All full-text original articles are obtained via open access or institutional subscription. All papers were in English, and none were excluded during the full-text screening stage. Ultimately, a total of 40 articles were gathered for the systematic review. There were 21 papers focused on *GUCY2C* (**Table 2**), eight papers on *GUCA2A* (**Table 3**), eight more papers on *GUCA2B* (**Table 4**), and finally, three papers that covered both *GUCA2A* and *GUCA2B*.

**Table 2:** GUCY2C-associated studies included in the systematic review.

Aim	Methods		Result	Ref.
	Data acquisition	Statistical analysis: Algorithms, R packages, etc.		
• Proposing a new method for predicting qRT-PCR efficiency by simulating the kinetics of PCR amplification used to evaluate the expression of GUCY2C mRNA in the blood samples of patients having colorectal cancer.	• 577 human blood samples	• qRT-PCR	• Correlation between the duration until reoccurrence and the longitudinal patterns in GUCY2C. expression was clinically meaningful	(30)
• Investigation of the impact of time, temperature, and ionic strength on the efficiency of radiolabeling and the attainable specific activity of a DOTA-conjugated high-lipophilic peptide, which incorporates three disulfide cyclization bonds.	• DOTA-MLN6907 as a GCC-specific peptide • ^68^Ga	• Reagents: Citric acid solution Sodium acetate buffers Ethanol solution •Classic and modified acetone approach • Purification and quality assessment following labeling using C-18 • Classic and alternative purification approaches • Detection of free thiol groups • Radiochemical purity and instrumentation	• The radiolabeling process achieved an efficiency exceeding 99%, with a specific activity surpassing 35 MB_q/nmole_ within time frame of under 30 minutes. • The fine-tuned parameters were suitable for implementing an automated 68Ge/68Ga generator and a fluid-handling system, enabling the clinical-scale production of the [68Ga] DOTA-MLN6907 peptide targeted to the GCC receptor. • The chemical properties unique to each peptide dictate the optimal conditions for radiolabeling, ensuring effective preparation of radiopharmaceuticals.	(31)
• Vaccine: Employing the CT26 murine colorectal tumor model to investigate the capacity of non-thermal plasmas to trigger immunogenic cell death *in vivo*	• Non-thermal plasmas • Cell lines CT26.WT CT26-GUCY2C • Balb/c mice	• *In vitro* plasma treatment • *In vivo* plasma treatment • Cell viability assay • ATP release assay • Detection of calreticulin exposed on the cell surface using fluorescence-based methods. • Anti-tumor vaccination assay • Staining tissue sections with H&E and evaluation of tissue damage. •Immunofluorescence staining of tumor tissue • ELISpot analysis	• Application of plasma treatment to subcutaneous tumors resulted in the release of danger signals and the attraction of antigen-presenting cells into the tumor microenvironment. • Enhanced T cell responses directed against the colorectal cancer-specific antigen GUCY2C were noted. • This research offers initial proof that non-thermal plasma genuinely triggers immunogenic cell death, underscoring its potential for practical application in cancer immunotherapy.	(32)
• Investigation of the effectiveness of a human-specific, GUCY2C-directed scFv as a foundation for constructing chimeric antigen receptors aimed at targeting metastases expressing human GUCY2C.	• Cell lines and reagents: CT26 β-galactosidase–expressing CT26.CL25 T84 SW480 CT26.CL25.hGUCY2C Luciferase-containing T84.fLuc 293FT 293F Metastatic tumor models: BALB/c mice NSG mice	• Cell culture • Murine CAR-T Cell Generation • Human CAR-T Cell Generation • CAR Surface Detection • Characterization of mouse T-cells through analysis of phenotypic markers, assessment of activation markers, and intracellular staining of cytokines. • Detection and analysis of activation markers in human T-cells, along with intracellular staining of cytokines. • T-Cell Cytotoxicity Assays	• Murine CAR-T cells directed toward human GUCY2C exhibited antigen-driven activation of T-cells, as evidenced by increased expression of activation markers, cytokine secretion, and selective elimination of GUCY2C-positive cancer cells *in vitro*. • CAR-T cells targeting GUCY2C conferred enduring safeguarding against lung metastases originating from murine colorectal cancer cells modified to express human GUCY2C, as demonstrated in a syngeneic mouse model. • Murine CAR-T cells targeting GUCY2C effectively identified and eliminated human colorectal cancer cells naturally expressing GUCY2C, leading to prolonged survival in a human xenograft model using immunodeficient mice.	(33)
• Evaluating the impact of cGMP-elevating substances on the process of tumorigenesis within the Apc^Min/+^ mouse model, a recognized model for intestinal cancer.	• Animal models of intestinal cancer male C57/BL6J-*Apc^Min/^* ^+^ mice and female C57/BL6J mice	• Histopathology • qRT-PCR	• Administering *Apc^Min^* ^/+^ mice with the receptor GCC agonist linaclotide or the PDE5 inhibitor sildenafil resulted in a noteworthy decrease in the polyp count per individual mouse. • Both PDE5 inhibitors and receptor GCC agonists demonstrate equal capability in inhibiting intestinal tumorigenesis in mice. • This study emphasizes the promising prospect of manipulating cGMP signaling as a strategy for chemopreventing colorectal cancer in human individuals with elevated risk.	(34)
• Evaluate the effects of TAK-264 in Asian patients with GI malignancies	• 12 patients aged ≥ 18 years diagnosed with GI carcinoma expressing GCC	• Patients’ eligibility test: immunohistochemistry • TAK-264 intravenous infusions as a human monoclonal anti-GCC antibody conjugated to monomethyl auristatin E • Evaluations: Dose-limiting toxicities Maximum tolerated dose Pharmacokinetics	• TAK-264 exhibited an acceptable safety record with modest effectiveness against tumors, aligning with findings from research involving patients with advanced gastrointestinal cancers in Western populations. • The amount of TAK-264 in the body rose in direct proportion to the dosage administered.	(35)
• Describing TCRs that identify both the intestinal epithelial cell receptor and the GUCY2C antigen associated with colorectal cancer. • Developing a framework for investigating mechanisms of self-antigen-specific tolerance.	• Animal models: BALB/c Gucy2c^−^/^−^ and Guc2yc^+^/^+^ and Rag1^−^/^−^ mice - Rag1−/− mice as the retrogenic model -	• Immunization with Ad5-GUCY2CECD • Immune responses: ELISpot or intracellular cytokine staining • GUCY2C-specific CD4+ T-cell isolation and TCR sequencing • Creating TCRs, generating retroviruses, and introducing TCRs into T-cells through transduction. • Surface markers and intracellular cytokine staining • Dual-color enzyme-linked ImmunoSpot assays • TCR avidity analysis	• Gucy2c^−^/^−^ mice lack self-tolerance toward the GUCY2C protein, leading to the production of immune responses targeting this protein. • GUCY2C-specific T-cell responses were observed upon immunization of Gucy2c^−^/^−^ mice carrying the TCR 4A or 5B variant. • GUCY2C-specific CD4+ T-cell responses, which were suppressed by tolerance in Gucy2c^+^/^+^ mice, were detected in Gucy2c^−^/^−^ mice that lacked tolerance mechanisms. • Collectively, these findings validate the effectiveness of TCR retrogenic mice generated through ex vivo TCR repertoire sequencing as a valuable model for investigating mechanisms related to GUCY2C-specific tolerance.	(36)
• Assess the potential therapeutic value of TAK-164, an advanced investigational ADC of the human anti-GCC monoclonal antibody linked to the potent DNA alkylator DGN549 through a peptide linker.	• HEK293 cell line • CB17 severe combined immunodeficient • Nude female mice	• Generation of anti-GCC antibodies and conjugation to DGN549 • Flow cytometry • *In vitro* cytotoxicity assay • Human xenograft tumor studies in mice • HEK-293 GCC cells or human primary tumors in DMEM were injected to mice • Pharmacokinetics analysis • Immunohistochemistry • ^89^Zr-immuno PET imaging	• Imaging investigations examined the uptake and functionality of TAK-164, revealing positive associations between tumor uptake and GCC expression, which were consistent with the observed antitumor effects. • TAK-164 exhibits significant efficacy across various GCC-positive tumors, even in cases where TAK-264, a GCC-targeted auristatin ADC, has proven ineffective. • A robust correlation exists among the uptake of 89Zr-labeled TAK-164, the extent of GCC expression, and notably, the response to TAK-164 treatment in both GCC-expressing xenografts and PHTX models.	(37)
• Investigation into the implications of mutant APC-β-catenin-TCF nuclear transcriptional re-programming on GUCY2C-cGMP signaling pathways.	• Human samples: tumors originated through the conventional pathway (Apc-β-catenin) • Animal models: *Apc^CKO^* mice contain a conditional knockout allele of APC • Cells: LS174T DLD1 HT29	• Cell culture •Immunofluorescence • Immunoblots • Messenger RNA analysis • New RNA synthesis	• In both human and mouse APC-dependent tumors, the loss of guanylin hormone expression occurs at the initial stages of transformation, while the GUCY2C receptor remains unaffected. • Broadening the mechanistic framework for colorectal cancer from being solely characterized by irrevocable mutations in APC and β-catenin, to encompass the loss of guanylin hormone, whose restoration and revival of GUCY2C signaling might hold the potential to deter tumorigenesis.	(38)
• Vaccine: Assessing the capacity of a chimeric adenoviral vector (Ad5.F35), created by combining the Ad5 capsid with the Ad35 fiber, to stimulate immune reactions targeting the tumor-associated antigen GUCY2C.	• BALB/cJ mice • Adenovirus vectors: Ad5.F35-GUCY2C-S1 • Vaccine: Adenovirus (Ad-GUCY2C) • Ad5-GUCY2C-S1, Ad5.F35-GUCY2C-S1, or Ad5.F35-GFP (control)	• Western blot • Quantifying T-cell responses by ELISpot • CRC cells *in vivo* tumor studies • Antibody neutralization assay • Ad5 neutralizing immunity studies • Biodistribution and toxicology study	• Ad5.F35-GUCY2C-S1 elicits a targeted immune response against GUCY2C, fostering antitumor immunity. • Antibody responses directed specifically against GUCY2C do not exhibit observable antitumor effects. • Both Ad5 and Ad5.F35 vaccines generated similar S1-specific CD4+ T-cell responses	(39)
• The objective is to explore the presence of circulating GCC mRNA and its connection with clinicopathological features, distant organ metastasis, and long-term survival among patients with stage I–III CRC.	• Circulating GCC mRNA of 160 CRC patient at stage I–III venous blood samples	• qRT-PCR	• The study demonstrated that circulating GCC mRNA serves as a dependable indicator for predicting metastasis and as a prognostic marker in patients with early-stage CRC. This underscores its potential to offer valuable guidance for initiating clinical interventions before tumor dissemination occurs.	(40)
• The promising therapeutic potential and tumor-specific effectiveness of PF-07062119, a CD3-bispecific T-cell engager designed to target tumors expressing GUCY2C. Its role also includes addressing immune evasion mechanisms employed by these tumors.	• Balb/c mice • OASIS 3.0 database for cell lines • PBMC Collection and Isolation of Human T cells for *in vitro* and *in vivo* studies • Whole blood of healthy donors for mononuclear cell and T cell isolation	• Generation of anti-GUCY2C antibodies • Mouse lymphoma 300.19 cells over-expressing human GUCY2C • Characterization of KRAS and BRAF mutational status of Colorectal Cancer CLX and PDX models • Immunohistochemistry • Human T Cell Adoptive Transfer Established Tumor Model • LS1034 Colorectal Orthotopic Tumor Model • CT26-mGUCY2C efficacy study in human CD3ϵ transgenic mice • Pharmacokinetic Measurements of GUCY2C(M)-CD3 in LS1034 Adoptive Transfer Model • Cell Culture • GUCY2C Receptor Density Measurements • Characterization of PF-07062119 Binding to human T cells and GUCY2C expressing tumor cells • Cytotoxic T Lymphocyte Assay • IFNγ Induced *In Vitro* by PF-07062119	• Tumors that express GUCY2C can be specifically targeted using an anti-GUCY2C/anti-CD3ϵ bispecific antibody, which results in the preferential distribution of the drug to the tumor sites. • F-07062119 demonstrated strong T-cell mediated activity *in vitro* and significant effectiveness *in vivo* across various human colorectal cancer xenograft models, including those with KRAS and BRAF mutations. Additionally, its efficacy was confirmed in an immunocompetent mouse model with syngeneic tumors. • The activity of PF-07062119 was amplified through synergistic effects when administered in combination with anti-PD-1/PD-L-1 treatment or alongside anti-angiogenic therapy.	(41)
• Explored the role of APC heterozygosity in mechanisms repressing hormone expression which could contribute to loss of heterozygosity	• Animal model as a monoallelic Apc loss Apc^min^/^+^ mice • Tissue specimens from two FAP patients as the human samples	• TCGA database • Immunofluorescence • Western blots • Messenger RNA analysis	• The presence of monoallelic APC loss in Apc^min^/^+^ mice did not result in any changes to hormone expression. • In patients with FAP, the loss of one allele of APC led to the expression of guanylin, whereas adenomas and cases with biallelic APC loss did not exhibit hormone expression. • Normal intestinal epithelial cells maintain uroguanylin and guanylin expression despite APC heterozygosity, but this expression is lost only after tumor initiation due to APC LOH.	(42)
• The objective is to assess the potential of PTGS2, JAG1, GUCY2C, and PGF-circulating RNA as biomarkers in metastatic CRC.	• 59 serum and blood samples of metastatic CRC patients • 35 patients received chemotherapy + antiangiogenic treatment and 24 patients received just chemotherapy. • Samples from 47 age- and sex-matched healthy controls were selected	• Digital PCR	• In terms of predicting treatment response, serum GUCY2C gene expression emerged as the most effective marker, demonstrating its utility in forecasting patient responses for both individuals receiving antiangiogenic treatment and those not undergoing such therapy. • Serum expression of GUCY2C and GUCY2C/PTGS2 demonstrated significant correlations with therapeutic response. However, none of the biomarkers showed correlations with overall survival or progression-free survival.	(43)
• The study involved the utilization of 89Zr-Df-IAB22M2C (also known as 89Zr-Df-Crefmirlimab), a human CD8-specific minibody, for the purpose of monitoring the infiltration of CD8+ T cells into tumors using positron emission tomography imaging.	• Whole blood of healthy donors for obtaining human T cells • Female NSG mice for xenograft studies	• Generation of anti-GUCY2C antibodies • PBMC collection and isolation and expansion of human T cells • LS1034 xenograft tumor model and bispecific antibody treatment • PET/CT Imaging and Tissue Assessments and analysis • CD8 Immunohistochemistry	• Substantial uptake of 89Zr-Df-IAB22M2C was seen in PF-07062119-treated tumors, significantly higher than controls, and response varied with PF-07062119 dose and treatment duration. • A moderate correlation was found between the uptake of radioactivity in tumor tissue and the density of CD8+ cells, underscoring the imaging agent’s utility for non-invasive evaluation of intra-tumoral CD8+ T cells and the mechanism of action of PF-07062119.	(44)
• Investigation into the potential application of oral dolcanatide, an uroguanylin analog designed for improved stability and extended presence in the gastrointestinal tract, to activate GUCY2C and induce cGMP production in the epithelial cells of the distal rectum among healthy volunteers.	• 27 screened healthy volunteers • Eight biopsies collected from the rectum during a flexible sigmoidoscopy procedure	• Direct comparison of cGMP levels versus placebo • Cyclic GMP quantification • Messenger RNA quantification	• While dolcanatide’s improved stability allows it to persist along the entire length of the small and large intestine, this alone is insufficient to effectively regulate GUCY2C throughout the colorectum and prevent tumorigenesis.	(45)
• To detect genes as signature related to CRC which can help to recognition of CRC in the early stage	• GEO database: GSE21510, GSE4107, GSE25071, GSE15781 and GSE8671	• GEO2R • STRING database	• GUCY2C was found as a suppressor gene in PPi networks of downregulated DEGs between CRC and normal samples in GSE datasets	(46)
• Vaccine: Investigating the immune response elicited by a Listeria monocytogenes (Lm) vaccine targeting the colorectal tumor antigen GUCY2C (Lm-GUCY2C).	• Lm-GUCY2C • Lm-LacZ • BALB/cJ mice • CT26 cell line for *in vivo* tumor studies	• live-attenuated double-deleted strain of Lm containing deletions in virulence factors internalin B and actA • IFNγ ELISpot Assay • MHC Class I Stability Assay • CAR Surface Detection • Tumor Studies	• Lm-GUCY2C induced strong CD8+ T-cell reactions against Lm-specific peptides, implying that the GUCY2C254-262 peptide might have a subordinate role compared to the Lm-derived peptides. • By introducing an amino acid substitution at a crucial anchoring site for H-2Kd binding, resulting in GUCY2CF255Y, the stability of the peptide complex improved significantly with H-2Kd. This modification effectively restored the immunogenicity of GUCY2C254-262 when integrated into the context of Lm vaccination.	(47)
• Vaccine: Investigating a prime-boost approach involving a chimeric adenoviral vector (Ad5.F35) engineered to overcome pre-existing immunity, followed by recombinant Lm to enhance immune response toward the gastrointestinal cancer antigen GUCY2C.	• Vaccines: Ad-GUCY2C • Adenovirus expressing mouse GUCY2C1-429 fused to the influenza HA107-119 CD4+ T-cell epitope • Lm-GUCY2C and Lm-LacZ • mouse macrophage cell line J774A.1 (ATCC) • BALB/cJ mice	• *In vitro* infections • Immunizations • Ad5-neutralizing immunity studies • IFNγ ELISpot assay • Intracellular cytokine staining • *In vivo* tumor studies • Safety studies • Blood chemistry and cytokine analyses • Western blot	• Immunization with both heterologous Ad-GUCY2C and Lm-GUCY2C enhances CD8+ T-cell responses specific to GUCY2C and boosts antitumor immune activity. • Previous exposure to Ad5 restricts the effectiveness of Ad5.F35+Lm immunization protocols, whereas prior Lm exposure does not impose such limitations. • Alterations in the qualitative characteristics of the CD8+ T-cell population after a prime-boost vaccination regimen. • Lm-GUCY2C could potentially be employed to enhance GUCY2C-specific immune responses in patients undergoing clinical trials with adenovirus-based GUCY2C vaccines, aiming to prevent or manage recurrent GI cancer.	(48)
• Investigating the process of GUCY2C ligand transcriptional suppression mediated by β-catenin/TCF signaling.	• RNA-seq gene expression data from normal mucosa n=51 and primary colon tumors n=380 from TCGA COAD/READ dataset • 4 unique conditional human colon cancer cell models of β-catenin/TCF signaling • Wild-type C57B/6(J) mice • Cell lines: DLD1, LS174T, TCF7L2, LS174T	• RNA sequencing analysis • luciferase reporters • Immunofluorescence • Immunoblots •RNA analysis and sequencing • Plasmids and Cloning • CRISPR Cell Line Generation • Lentiviral Production and Transduction • Chromatin Immunoprecipitation and sequencing • TCGA dataset analysis	• RNA sequencing analyses uncover GUCY2C hormones as among the most responsive targets of β-catenin/TCF signaling, indicative of transcriptional downregulation. • The GUCY2C hormones are situated within a distinct genomic region, featuring a novel locus control region positioned upstream of the guanylin promoter. This regulatory element plays a role in orchestrating the simultaneous suppression of both genes. • Using CRISPR epigenome editing to target this region led to the restoration of GUCY2C ligand expression, effectively overcoming the gene inactivation caused by mutant β-catenin/TCF signaling.	(49)
• Evaluating the safety and tolerability profile of TAK-164, an experimental antibody-drug conjugate targeting GCC	• 31 patients with GCC-positive, advanced gastrointestinal cancers	• Intravenous TAK-164 on day 1 of 21-day cycles via a Bayesian model	• Except cycle 1, dose-limiting toxicities evaluation in subsequent cycles dose-limiting treatment-emergent adverse events such as grade 3 pyrexia, grade 5 hepatic failure, decline in platelet and neutrophil count in patents were observed • A single patient (at a dose of 0.008 mg/kg) who exhibited elevated initial GCC expression demonstrated a preliminary but unconfirmed partial response. • TAK-164 exhibited a controlled and well-handled safety profile when administered at a dose of 0.064 mg/kg. • The Recommended Phase 2 Dose (RP2D) of 0.064 mg/kg was deemed inadequate to achieve clinical benefit, consequently leading to the decision of not pursuing further clinical development.	(50)

qRT-PCR, Quantitative Real-Time Polymerase Chain Reaction; GUCY2C, Guanylate Cyclase 2C;DOTA, Dodecane Tetraacetic Acid; ScFv, Single-chain variable fragments; H&E, haematoxylin and eosin; TCR, T cell receptor; CAR, Chimeric Antigen Receptors; APC, Adenomatous Polyposis Coli; PDE, phosphodiesterase; GC-C, Guanylate Cyclase-C; TCR, T cell receptor; ELISpot, enzyme-linked immunosorbent spot; DMEM, Dulbecco’s Modified Eagle Medium; PET, positron emission tomography; PHTX, Primary Human Tumor Xenograft; TCF, T Cell Factor; ADC, antibody-dug conjugate; CRC, colorectal cancer; Ad, Adenovirus; GFP, Green Fluorescent Protein; PBMC, Peripheral Blood Mononuclear Cells; CLX, cell line xenograft; PDX, patient-derived xenograft; TCGA, The Cancer Genome Atlas; FAP, Familial Adenomatous Polyposis; LOH, loss of heterozygosity; NSG, NOD-scid IL2Rγnull; GEO, Gene Expression Omnibus; STRING, Search Tool for the Retrieval of Interacting Genes/Proteins; PPi, Protein–protein interaction; DEGs, Differentially Expressed Genes; Lm, Listeria Monocytogenes; ATCC, American Type Culture Collection; MHC, Histocompatibility Complex; GI, Gastrointestinal; CRISPR, clustered regularly interspaced short palindromic repeats; RP2D, Recommended Phase II Dose.

**Table 3:** GUCY2A-associated studies included in the systematic review.

Aim	Methods		Result(s)	Ref.
	Data acquisition	Statistical analysis: Algorithms, R packages, etc.		
Exploring the potential significance of the GUCA2A-GUCY2C axis and its viability as a target in tumors originating from the SA and MSI pathways.	• TCGA database • GEO database • Human tumor tissues and histological interpretation • Mouse tissues • RNA isolation • Quantitative reverse-transcription polymerase chain reaction • Immunoblot analysis	• GraphPad Prism	• Guanylin hormone expression was omitted in TAs, SAs, and MSI tumors compared to their corresponding normal adjacent tissues. • Silence GUCA2A in pathophysiology and utilize oral hormone replacement to restore GUCY2C signaling, preventing MSI tumors.	(51)
To pinpoint a gene with notable clinical relevance for CRC diagnosis, treatment, and prognosis prediction.	• GEO database • WGCNA • RRA Analysis • Survival Analysis • qRT-PCR	• Limma package • RobustRankAggreg • WGCNA package	• GUCA2A was identified as a hubgene in CRC. • GUCA2A expression was significantly correlated with the OS of CRC patients. • qPCR analysis showed that GUCA2A expression in tumor and metastatic tissues was significantly low compared with adjacent normal tissues. • GUCA2A has a potential diagnostic value for CRC patients with 80.6% sensitivity and 83.5% AUC.	(52)
To pinpoint fundamental genes linked to CRC.	• GEO database • GEPIA database • Limma package • Enrichment analysis • qRT-PCR • STRING database	• clusterProfile package • Limma package • VennDiagram package	• GUCA2A identified as a hub gene in CRC. • Expression levels of GUCA2A were not correlated with the overall survival in CRC patients. • qRT-PCR showed that there was no significant differential expression of GUCA2A between CRC tissues and normal colorectal tissues.	(16)
To explore potential gene targets for the diagnosis and treatment of PCRC	• GEO database • TCGA database • DAVID database • Enrichment analysis • STRING database • cBioPortal database • Xena database • GEPIA database • Kaplan-Meier plotter • comparative toxicogenomics database	• GEO2R	• GUCA2A was identified as a hub gene in PCRC. • Expression level of GUCA2A was not correlated with the overall survival of PCRC patients.	(53)
To extract the gene expression profile data of colon cancer from TCGA database, and explore the potential utility of the TMB in immunotherapy and individualized medication.	• TCGA database • TMB value estimation • Relationship between TMB value and overall survival • Correlation between TMB value and clinicopathological features • Relationship between TMB and differentially-expressed genes	• CRAN package • Limma package • Pheatmap package • clusterProfiler package • org.Hs.eg.db package • ggplot2 package • CIBER-SORT analysis platform • Survival package	• GUCA2A is positively correlated with CRC patients’ survival	(54)
Assess gender-related distinctions in the transcriptome of both non-tumor colon epithelium and CRC	• TCGA database • RNA isolation • qPCR • feature selection • machine learning classification • Overall survival analysis	• Bioconductor package	• GUCA2A identified as a CRC prognostic biomarker in males	(55)
To identify CRC-associated genes	• GEO database • Identifcation of common DEGs in CRC and diferentially expressed miRNAs • STRING database • Identifcation of CRC−associated core genes	• Limma package	GUCA2A identified as a core gene in CRC.	(56)
Discover potential novel biomarkers for CRC and gain deeper insights into the molecular pathways contributing to CRC development.	• GEO database • Processing of lncRNA expression profiles • WGCNA and the identification of modules	• GEOquery package • Limma package • WGCNA package	• GUCA2A identified as a hub gen in CRC.	(57)
To mine the GEO datasets associated with CRC studies and identify potential targets correlated with CRC pathogenesis.	• GEO database • Screening common DEGs • Kaplan–Meier Survival Analysis of Patients with CRC	• GREIN-iLINCS	• GUCA2A was identified in a key sub-network in CRC. • Expression level of GUCA2A was not correlated with the overall survival of CRC patients.	(58)
To discover key pathways and genes implicated in the onset, development, and adverse prognosis of CRC.	• GEO database • GEPIA database • UALCAN database • OncoLnc database • DAVID database • STRING database	• GEO2R	• GUCA2A identified as a hub gene in CRC. • GUCA2A demonstrated a significant association with lower survival rates.	(59)
To identify central genes linked to colorectal adenocarcinoma and subsequently assess their prognostic relevance.	• GEO database • TCGA database • WGCNA analysis • Enrichment analysis • STRING database • HPA database	• Limma package	• GUCA2A identified as a hub gene in CRC. • CLCA1, CLCA4, and GUCA2A were identified as a 3-Gene Signature in CRC • Protein levels of GUCA2A are significantly lower than normal tissues.	(60)

GUCA2A, Guanylyl cyclase-activating protein 2A; TCGA, The Cancer Genome Atlas; GEO, Gene Expression Omnibus; TA, tubular adenomas; SA, serrated adenoma; MSI, microsatellite instability; CRC, colorectal cancer; WGCNA, Weighted gene correlation network analysis; RRA, Rapid Risk Assessment; OS, overall survival; qPCR, quantitative polymerase chain reaction; AUC, Area under the Curve; PCRC, Primary colorectal cancer; GEPIA, Gene Expression Profiling Interactive Analysis; STRING, Search Tool for the Retrieval of Interacting Genes; DEGs, Differentially Expressed Genes; DAVID, database for annotation; visualization and integrated discovery; CLCA, Chloride channel accessory; HPA, The Human Protein Atlas.

**Table 4:** GUCY2B-associated studies included in the systematic review.

Aim	Methods		Result	Ref.
	Data acquisition	Statistical analysis: Algorithms, R packages, etc.		
suggesting a diagnostic method based on KRAS mutation and gene expression analysis that may be regularly used in clinics to choose the best course of action for each patient.	• Twenty-four patients who underwent surgery in the years 2013 or 2014 at the two University hospitals “Kaspela” and “St. George”	• DNA Isolation • RNA Isolation • qPCR	• GUCA2B is the gene with the most severe downregulation. • GUCA2B exhibits the most pronounced transcriptional downregulation.	(61)
In order to outline the indicators and transcriptional conditions, the goal is to recognize a colonic epithelial cell and reveal essential factors that lead to barrier dysfunction in inflammatory bowel disease.	• Isolation of epithelial cells from patients with IBD biopsies • GEO database: GSE116222 • Github • ProteomeXchange Consortium	• Isolation of epithelial cells from patient biopsies • Droplet-based single-cell RNA sequencing • Plate-based scRNAseq • RT-PCR • Proteomic analysis of BEST4/OTOP2 cell population • Animal Experiment • SEM and TEM • Organoid culture *Computational analysis: Cell Ranger • Crypt-axis score • Semisupervised clustering of public scRNA-seq data • TCGA • Cluster marker and differentially expressed gene identification • Microarray analysis • Ontology enrichment analysis • Smart-seq2 scRNA-seq data processing and analysis • Proteomics data analysis • BEST4/OTOP2 cell marker overlap • Trajectory and pseudo-time analysis • Analysis of tissue-specific • expression of GWAS loci	• GUCA2B’s peptide uroguanylin activates GC-C and cyclic GMP in epithelial cells containing metallothionein genes, guarding against free radicals and transporting and storing metals. • BEST4/OTOP2 cell which expresses uroguanylin, identified. According to the research, IBD and colorectal cancer cases have lower levels of uroguanylin-producing colonic epithelial cells.	(62)
Using bioinformatics, this study aims to investigate possible gene targets for the detection and therapy of PCRC	• GEO database: 1. GSE81558 dataset • GO database • KEGG database • DAVID	• GEO2R • Limma • GEOquery • Metascape • GSEA • STRING • Molecular Complex Detection • cytoHubba • cBioPortal • GEPIA • Kaplan-Meier plotter • CTD	• The expression of GUCA2B did not have any statistically significant effects on OS. • GUCA2B is a key gene among DEGs.	(53)
The current study aims to apply bioinformatics to discover the PCRC hub genes and to confirm their impact on patients’ overall survival based on clinical data.	• GEO database: GSE81558, GSE41258 and GSE81558 • DAVID • KEGG database	• Pearson’s correlation test • principal component analysis • GEO2R • Limma package • GEOquery • SangerBox • online Venn tool • STRING • Gene Ontology analysis • Molecular Complex Detection tool • Cytoscape • cytoHubba • cBioPortal • Coexpedia • GEPIA • SPSS software • RT-qPCR assay	• GUCA2B is a key gene among 10 hub genes. • The expression of GUCA2B did not show a statistically significant impact on overall survival	(63)
This study employed an integrated analysis, combining gene expression patterns from four microarray datasets in GEO with miRNA expression profiles. The analysis aimed to identify genes associated with CRC through microarray data analysis.	• GEO database: GSE37182, GSE25070, GSE10950 and GSE113513 datasets • miRNA platform: • GSE115513 and GSE30454 • DAVID • KEGG database	• Limma package • Venn diagrams website • Gene ontology analysis • STRING website • Cytoscape program	• GUCA2B is a key gene among 10 hub genes. • GUCA2B with a score of 8 and standing in 4th rank between other hub genes and interacting with other nine hub genes suggesting their crucial role in CRC progression	(56)
Through an in-depth examination of the data in TCGA, the current work aims to acquire an understanding of the processes regulating B4GALNT2 expression and its relationship to cancer	• Oncolnc website • Gene expression data of 626 colorectal adenocarcinomas (COADREAD) from TCGA • Gene methylation data of 288 tumor samples were downloaded from TCGA • CSmiRTar website	• Kaplan–Meier survival curve • Smartapp tool • genecards.org”	• A larger molecular profile, including GUCA2B, is linked to high B4GALNT2 expression as a reliable indicator of a favorable prognosis in CRC.	(64)
Suggesting chemically altering uroguanylin, which is expressed in tumors that have metastatic CRC, to encourage its anchoring to SNs (UroGm-SNs).		• Preparation of UroGm-SNs • Physicochemical characterization • Morphological examination • Ligand density calculation • Preparation of dual-loaded SNs • Cell viability studies • Cellular internalization studies • Colony forming assays • *In vivo* effectiveness of UroGm-Etp-SNs in mice harboring SW620 xenografts.	• UroGm was effectively engineered to anchor onto the surface of nanoparticles, maintaining its therapeutic attributes without compromise.	(65)
The primary objective of the recent study was to investigate possible transcriptome biomarkers or treatment targets of CRC.	• GEO database: 2. GSE89393 dataset • preservation analysis: 3. GSE41328, GSE54986, GSE81582, GSE100179, GSE113513, and GSE137327 datasets • RNA-seq data: 4. Colon Adenocarcinoma (COAD) patients were obtained from the TCGA database • UCSC Cancer Browser • TargetScan databases • GSE180202 dataset	• edgeR package • CluePedia • WGCNA • TOM • dissimilarity TOM (dissTOM) • Module Eigengene • GEPIA • RT-qPCR • Shapiro-Wilk normality test • Venny software • STRING website	• The brown module correlated positively with CRC in WGCNA. • CRC tissues showed lower GUCA2B expression by RT-qPCR. • GUCA2B is a key gene among DEGs. • The top miRNAs associated with GUCA2B were found. • According to ROC studies, GUCA2B has a strong diagnostic performance for CRC.	(66)
To find possible targets linked to CRC pathogenesis, we mined the GEO datasets connected to CRC research.	• GEO database: 5. GSE50760 and GSE104178 datasets • KEGG database	• GREIN-iLINCS online analysis tool • EnrichR • STRING tool • miRDB • Cytohubba tool • Cytoscape software • Kaplan Meier plotter • GEPIA tool	• There was no correlation between the expression levels of GUCA2B with the overall survival of CRC patients. • GUCA2B is a key gene among DEGs.	(58)
This study investigated the association between ICIs in CRC and missense mutations in DNAH7, the gene encoding the axonemal dynein heavy chain.	• A clinical cohort (n=690) • TCGA database • GO database • KEGG database • MSIgDB database	• VarScan software • TCGAbiolinks package • GenePattern5, GISTIC2.0 • Wilcoxon rank-sum test • DESeq2 package • Gene Ontology analysis • ClusterProfiler R software package • GSEA • ESTIMATE analysis • The Mann-Whitney U test • STRING online tool • Cytoscape (V3.7.2) • Maximal Clique Centrality (MCC) algorithm • cytoHubba	• GUCA2B emerges as a prominent key gene linked to the DNAH7 mutation.	(67)
To explore the molecular signatures causing CRC as receptors and drug agents as inhibitors by using integrated statistics and bioinformatics approaches.	• GEO database: 6. GSE9348, GSE110224, GSE23878, and GSE35279 datasets.	• LIMMA package • STRING database • GEPIA • TIMER • MethSurv • Enrichr • DisGeNET • miRTarBase • DSigDB	• GUCA2B is a key gene among DEGs. • Downregulation of GUCA2B in COAD and READ. • GUCA2B gene is significantly methylated at the CpG site	(68)

qPCR, quantitative polymerase chain reaction; GUCA2B, Guanylate Cyclase Activator 2B; GEO, Gene Expression Omnibus; RT-PCR, Reverse transcription polymerase chain reaction; SEM, Scanning electron microscopy; TEM, Transmission electron microscopy; GC-C, Guanylate cyclase-C; IBD, Inflammatory bowel disease; TCGA, The Cancer Genome Atlas; KEGG, Kyoto Encyclopedia of Genes and Genomes; DAVID, database for annotation; visualization and integrated discovery; GSEA, Gene set enrichment analysis; STRING, Search Tool for the Retrieval of Interacting Genes; GEPIA, Gene Expression Profiling Interactive Analysis; CTD, The comparative toxicogenomics database; CRC, colorectal cancer; SNs, sphingomyelin nanosystems; UroGm, uroguanylin expressed in metastatic colorectal cancer tumors; WGCNA, Weighted gene correlation network analysis; TOM, Topological Overlap Matrix; DEGs, Differentially Expressed Genes; ROS, Receiver operating characteristic; READ, Rectal Adenocarcinoma.

### 3.1 GUCY2C

**Figure 3:**
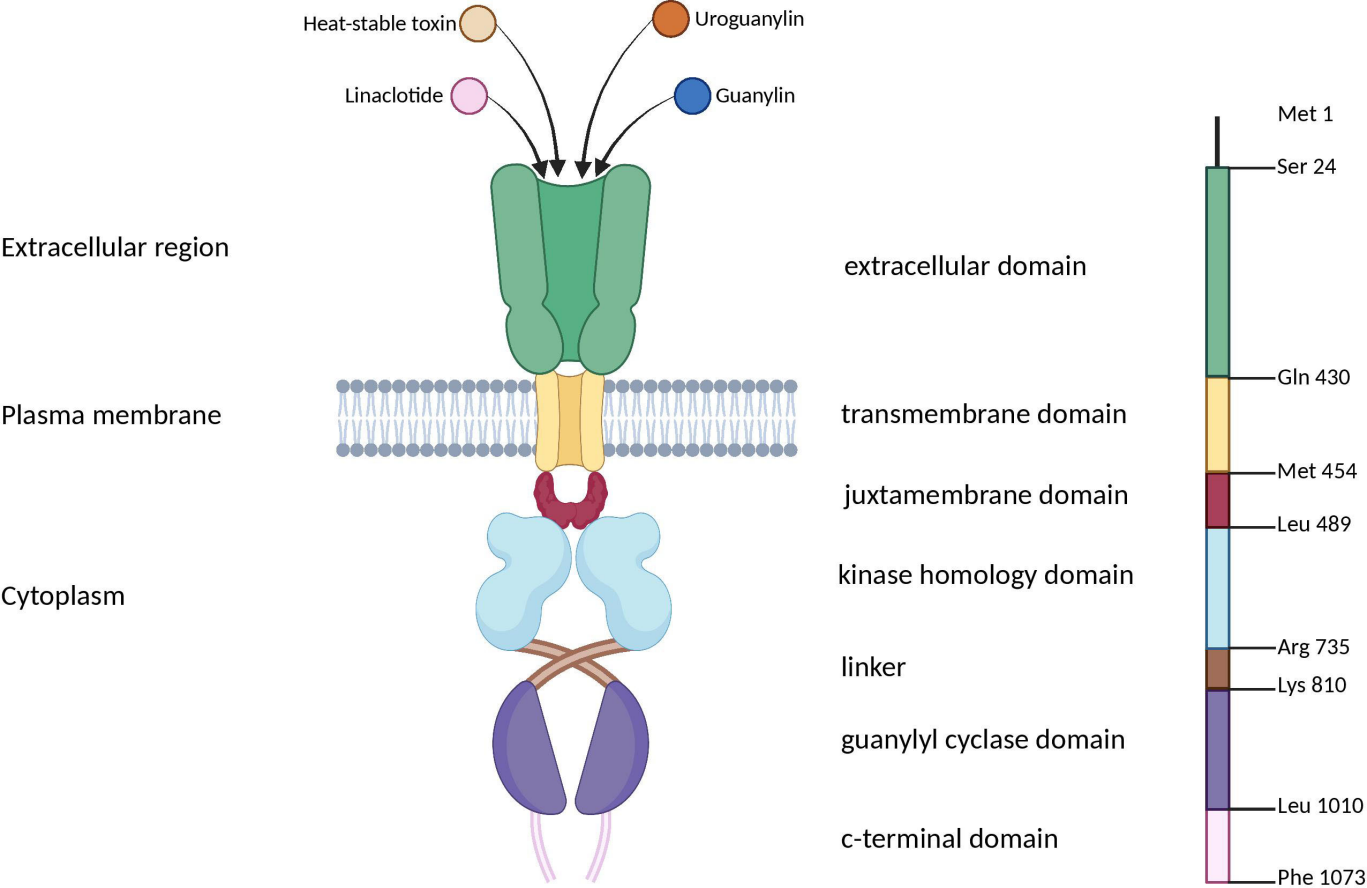
Schematic representation of domain organization of the guanylyl cyclase GC-C receptor. GC-C is predicted to be a transmembrane receptor homodimer with seven functional domains. These domains include the extracellular domain responsible for binding peptide ligands such as STs, guanylin, uroguanylin, and the FDA-approved ST analog, Linaclotide. Additionally, there is a transmembrane domain, a juxta membrane domain, a kinase-homology domain, and a linker region that may facilitate catalytic subunit dimerization and regulate its function. GC-C, Guanylate Cyclase-C; ST, heat-stable enterotoxins. Created with BioRender.com.

The *GUCY2C* gene encodes the GC-C receptor, a membrane protein composed of various domains, including extracellular binding, transmembrane, juxta-membrane, kinase homology, linker, guanylyl cyclase, and C-terminal domains (**Figure 3**) (69, 70). GC-C is triggered by the guanylin family of peptides, which consists of endogenous peptides, including GN, UG, and lymphoguanylin, and an exogenous peptide toxin produced by enteric bacteria (71, 72).

Growing evidence suggests a close association between CRC and dysbiosis of gut microbiota. Recent studies have shed light on the specific roles of intestinal microorganisms in initiating and facilitating the development of CRC. Notably, our review demonstrates that ETEC produces a substance called ST, which binds to the GC-C receptor with high affinity. ETEC is an important cause of diarrheal disease, particularly in low- and middle-income countries (73). Conversely, the incidence of CRC in developed countries is up to ten times higher than in underdeveloped countries (74). Intriguingly, an inverse epidemiological correlation exists between diarrheal diseases caused by ST-producing ETEC and colorectal cancer. This inverse correlation may be attributed to the involvement of *GUCY2C* as a tumor suppressor in CRC pathophysiology, particularly in developing countries. A recent study in mice has revealed that chronic colonization with ST-producing Escherichia coli counters the development of colorectal tumors. These findings suggest that bacterially produced ST reinstates *GUYC2C* signaling, which in turn opposes the tumor transformation (75). Additionally, more research is needed to investigate the potential of using ST as a possible prophylactic or therapeutic approach for preventing or treating CRC. These studies could pave the way for developing novel strategies for CRC prevention and treatment.

**Figure 4:**
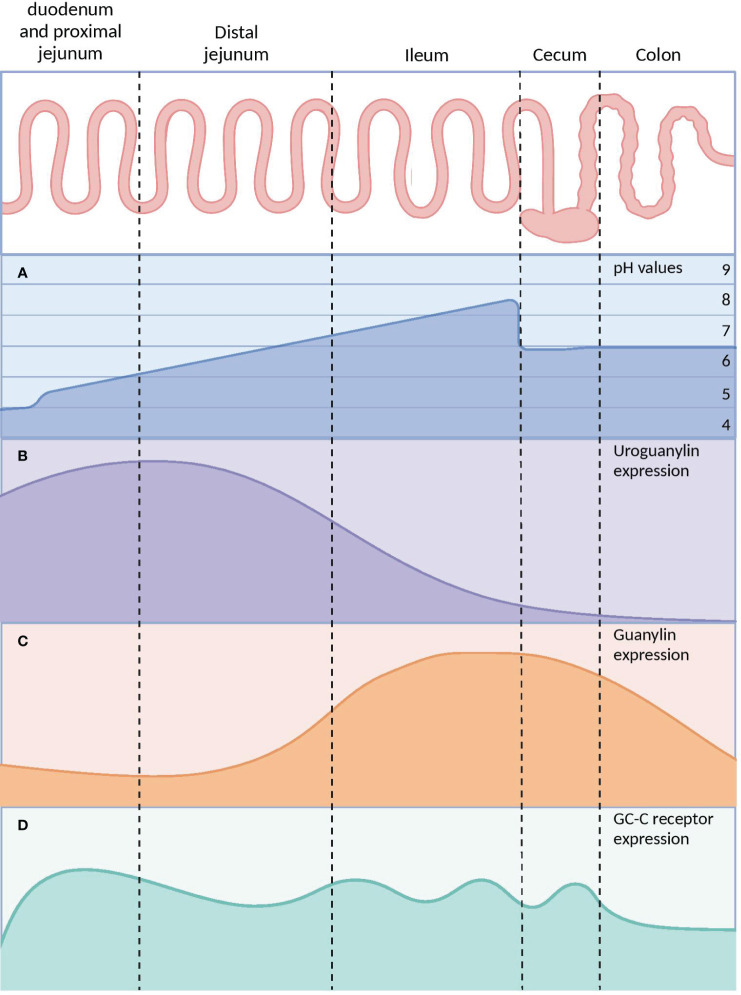
The differences in pH and GC-C signaling axis parts throughout the gastrointestinal tract. **(A)** The pH of the small intestine increases gradually while the pH of the caecum decreases due to microorganism populations. **(B)** UG expression is at its maximum in the distal jejunum and at its lowest in the colon. **(C)** GN concentrations rise along the distal small intestine, peak in the caecum, and then drop rapidly in the distal colon. **(D)** The expression of GC-C receptors is constant throughout the intestine. GC-C, Guanylate Cyclase-C; UG, Uroguanylin; GN, Guanylin. Created with BioRender.com.

Moreover, the activation of the GC-C signaling axis is pH-dependent, meaning that different parts of the gastrointestinal (GI) tract have different regulatory effects (28, 29). The distribution of GC-C receptors throughout the GI tract is widespread, but their activation patterns depend on the location and type of activator. In the upper small intestine, GC-C receptors are more strongly activated at lower pH levels (~ pH 5.5) by UG. On the other hand, in the lower small intestine and colorectum, these receptors are activated at higher pH levels (~ pH 8.0) by GN (**Figure 4**) (76, 77).

**Figure 5:**
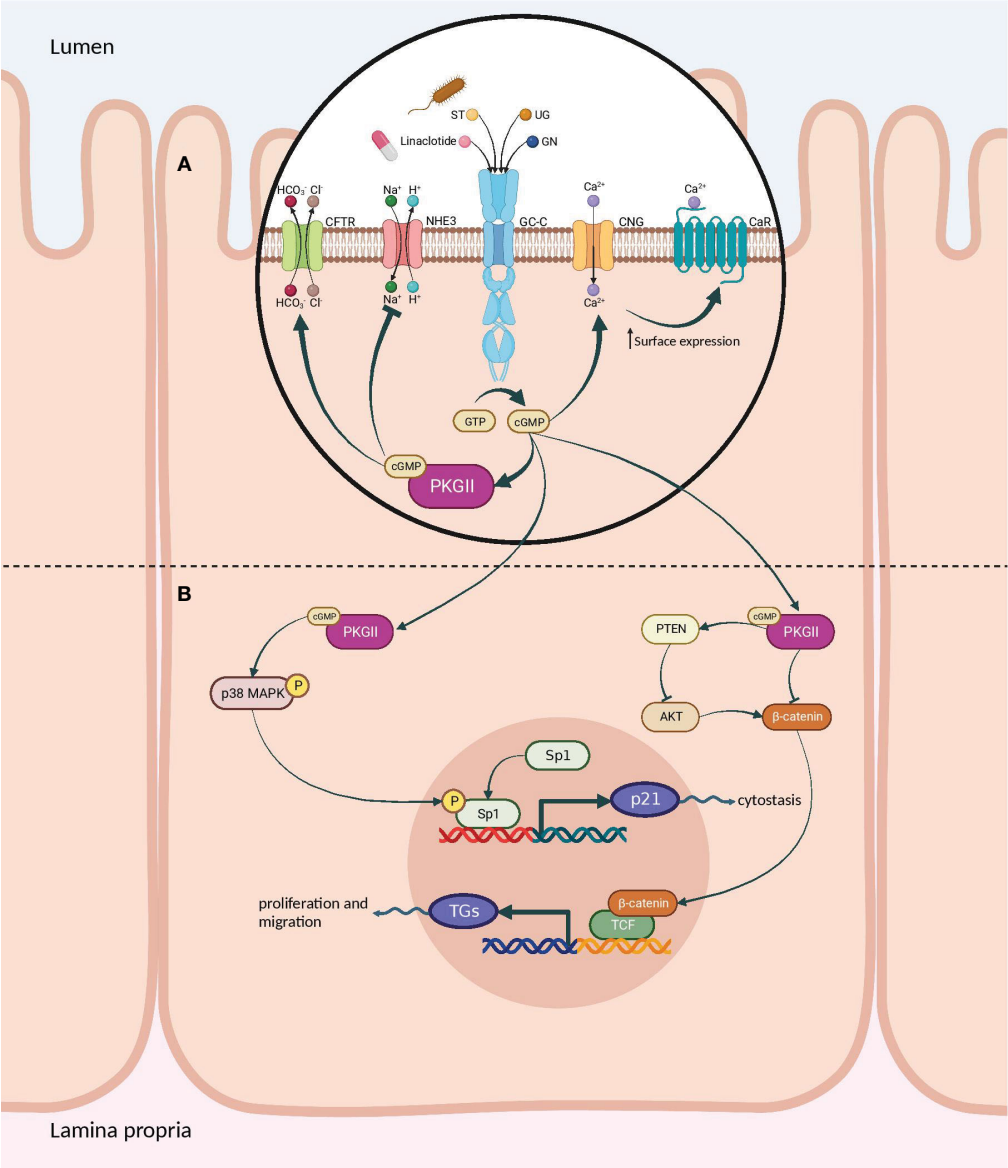
Signaling pathways of the GC-C/cGMP axis that regulate fluid-ion homeostasis and cellular proliferation in the intestine. **(A)** The binding of ligands to GC-C catalyzes the formation of cGMP from GTP. Increased intracellular cGMP levels result in the activation of cGMP-dependent PKGII. Reduced intestinal sodium absorption is caused by PKGII-mediated inhibitory phosphorylation of NHE3. PKGII phosphorylated and activated the CFTR anion channel, increasing intestinal chloride and water secretion. Increased cGMP activates CNG ion channels, promoting Ca2^+^influx, which recruits CaR to the plasma membrane. **(B)** Cyclic GMP production activates PKGII and p38 MAPK, resulting in phosphorylation of the Sp1 transcription factor. Sp1 upregulates the expression of p21 and mediates cytostasis. PKGII-mediated signaling opposes pro-survival and pro-proliferative phenotypes mediated by the β-catenin/TCF and Akt pathways. GC-C, Guanylate Cyclase-C; PKGII, Protein kinase G II; Protein kinase; NHE3, Na^+^/H^+^ exchanger isoform 3; CFTR, Cystic fibrosis transmembrane conductance regulator; CNG, Cyclic nucleotide-gated; CaR, calcium-sensing G-protein coupled receptors; MAPK, Mitogen-activated protein kinases; TCF, T cell factor. Created with BioRender.com.

Upon binding of agonist peptides to the extracellular domain of the GC-C receptor, the intracellular catalytic domain converts guanosine triphosphate into cyclic guanosine monophosphate (cGMP). This second messenger then activates cGMP-dependent protein kinases G (PKG), cyclic-nucleotide-gated (CNG) channels, and cGMP-regulated cyclic-nucleotide phosphodiesterase (78). In intestinal cells, protein kinase G II (PKGII) phosphorylates the cystic fibrosis transmembrane conductance regulator (CFTR), leading to increased efflux of chloride (Cl^−^) and bicarbonate (HCO^−^) ions from intestinal cells into the lumen. The resulting anion efflux causes a net osmotic increase, driving water into the GI tract and promoting fluid secretion (**Figure 5A**) (78, 79).

GC-C also controls the balance between proliferation and differentiation by increasing p21 expression under normal physiological circumstances (77, 80). It has been shown that GC-C mediates antitumorigenic processes in addition to p21-mediated cytostasis. One prominent instance is the GC-C signaling-mediated attenuation of β-catenin-mediated TCF transcriptional activity. PKGII-mediated signaling opposes β-catenin/TCF-mediated proliferative and promigratory phenotypes (81, 82). β-catenin/TCF signaling, in turn, suppresses the GC-C axis by inhibiting the transcription of its ligands, GN and UG. Additionally, GC-C blocks PTEN-mediated pro-tumorigenic Akt signaling (**Figure 5B**) (78, 83–85). As a tumor suppressor, GC-C regulates the migration and differentiation of stem cells at the base of intestinal crypts into enterocytes and other cell types (23). In fact, inhibiting the GC-C axis results in hyper-proliferation, hyperplasia of proliferating crypts, accelerated migration, diminished differentiation along the secretory lineage, and decreased apoptosis (77).

Another noteworthy aspect of GC-C is its potential as a tumor biomarker for CRC detection. While guanylyl cyclase family members are typically only expressed in normal intestinal cells, GC-C is found in approximately 95% of colorectal and some other gastrointestinal cancers, such as pancreatic tumors. In contrast, GC-C expression is rare in non-intestinal tissues and tumors. This suggests that GC-C could be a valuable and novel biomarker for identifying CRC (17, 86, 87).

In a study conducted by Jimenez-Luna et al. (43), the potential of *GUCY2C*, *PTGS2*, *JAG1*, and *PGF* circulating RNAs as biomarkers in metastatic CRC (mCRC) was investigated. The researchers collected 59 serum and blood samples from mCRC patients, divided into two groups: one receiving chemotherapy plus antiangiogenic treatment and the other receiving only chemotherapy. Additionally, 47 healthy control samples were included. The samples were then analyzed using digital polymerase chain reaction (PCR). The study revealed a significant correlation between *GUCY2C* and *GUCY2C/PTGS2* expression in the bloodstream and the response to anti-angiogenic agents. These results suggest that evaluating genes involved in the process of angiogenesis could serve as a promising non-invasive diagnostic tool for metastatic colorectal cancer and predict its response to anti-angiogenic therapy (43) (**Table 2**).

Blomain et al. (38) contributed significantly to understanding intestinal tumorigenesis by uncovering the crucial role of the GN hormone and GC-C receptor signaling in tumor initiation and progression. Specifically, their study revealed that the loss of GN hormone expression, but not the GC-C receptor, occurs at the earliest stages of adenomatous polyposis coli (APC)-dependent tumor transformation in both humans and mice. This loss of GN expression results from mutant APC-β-catenin-TCF transcriptional regulation, which suppresses GC-C signaling and perturbs intestinal homeostatic mechanisms, contributing to tumor progression. The authors proposed that the replacement and reconstitution of GC-C signaling could prevent tumorigenesis by restoring the GN hormone expression (38). This finding provides a potential therapeutic target for preventing the development and progression of intestinal tumors.

Recent studies have focused on the extensive utilization of *GUCY2C* biology in experimental cancer immunotherapy, including developing vaccines, immunotoxins, and chimeric antigen receptor (CAR) T cells (88). Developing cancer vaccines that can activate the immune system to identify and destroy cancer cells holds immense potential (89). One approach that has recently piqued interest is targeting the GC-C receptor to create effective cancer vaccines. For example, the promising results in preclinical and clinical trials suggest that GC-C-based therapies could effectively treat cancer. In the study by Flickinger et al. (48), a prime-boost strategy was investigated that involved the use of a chimeric adenoviral vector (Ad5.F35) that is resistant to pre-existing immunity, followed by recombinant Listeria monocytogenes (Lm) to amplify immunity to the GI cancer antigen GUCY2C. It was found that the combination of Ad5.F35 and Lm-GUCY2C enhanced the quantity, avidity, polyfunctionality, and antitumor efficacy of GUCY2C-specific effector CD8+ T cells in mice. The results suggest that Lm-GUCY2C could be used to increase GUCY2C-specific immunity in patients who are given adenovirus-based GUCY2C vaccines that are currently being tested in clinical trials to prevent or treat recurrent GI cancer (48).

In another study, Lin et al. (32) demonstrated the potential of non-thermal, atmospheric pressure plasma (NTP) in inducing immunogenic cell death in an animal model of CRC. The study included assessments of cell viability, anti-tumor vaccination assays, and ELISpot analysis. The findings suggest that NTP treatment can enhance T-cell responses targeting the CRC-specific antigen GC-C, potentially enhancing the immune system’s ability to recognize and eliminate cancer cells. The study sheds light on the possible role of non-thermal plasma in stimulating immunogenic cell death for future clinical applications in cancer immunotherapy and vaccine manufacturing, particularly for CRC.

CAR T-cell therapy is a promising treatment method by engineering T cells to express CARs that can target cancer cells. By doing this, the immune system can selectively attack and eliminate tumors (90). A highly optimistic target for this therapy is the GC-C receptor. In an investigation, Magee et al. (33) engineered a human-specific single-chain variable fragment (scFv) directed toward GC-C to engineer CAR-T cells for the treatment of colorectal cancer metastasis. The CAR-T cells were tested in preclinical murine models and provided long-term protection against murine colorectal cancer cells expressing human *GUCY2C* lung metastases. The study also demonstrated the ability of the CAR-T cells to recognize and kill human colorectal cancer cells expressing *GUCY2C* in a xenograft model in immunodeficient mice. These findings suggest the potential of human *GUCY2C*-specific CAR-T cell therapy for the treatment of metastatic colorectal cancer expressing *GUCY2C* (33). GUCY2C-targeted CAR T cells could offer a novel and effective strategy for treating CRC, particularly those in advanced stages of cancer or refractory to conventional therapies.

### 3.2 GUCA2A

**Figure 6:**
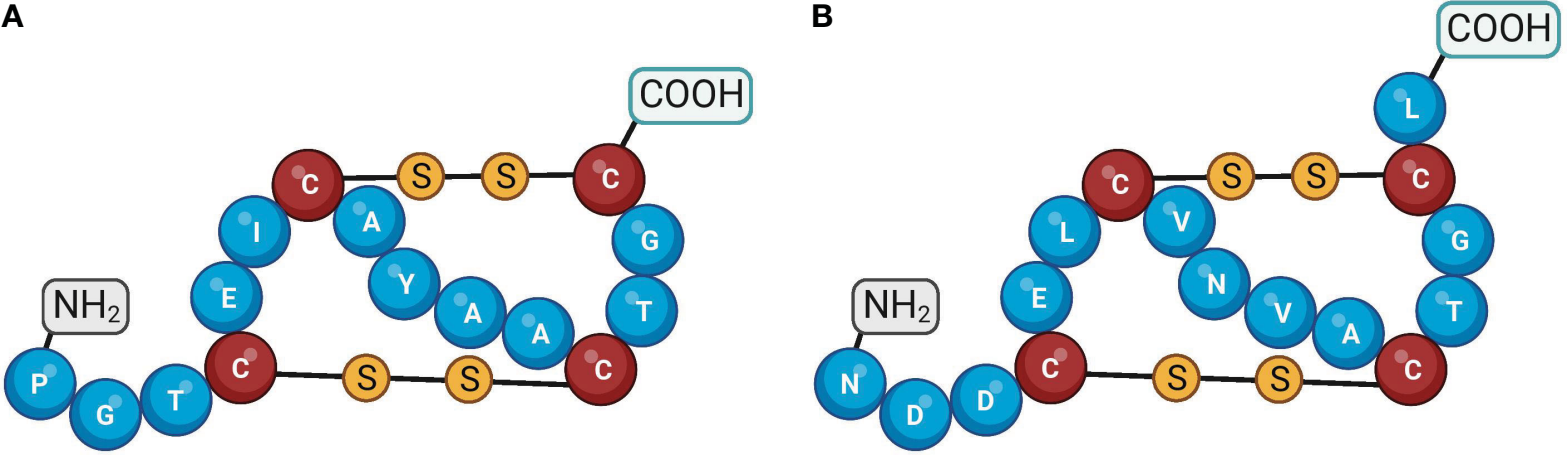
Amino acid structures of guanylin and uroguanylin. Guanylin **(A)** and uroguanylin **(B)** The cysteine residues are shown by the different colors of the amino acid and by the disulfide bonds. Created with BioRender.com.

The Guanylate Cyclase Activator 2A (*GUCA2A*) gene encodes Guanylin (GN), which is a bioactive peptide synthesized in the intestinal mucosa and acts as an endocrine ligand for GC-C. GN serves crucial functions in maintaining intestinal fluid homeostasis and preserving gut physiology (91). The mature form of guanylin consists of 15 amino acids derived from the C-terminus of a longer pre-proguanylin peptide. This longer peptide includes a signal sequence and a proguanylin sequence span residues 1-21 and 22-115, respectively (92). Although some studies indicate that proguanylin is the primary form of the peptide that is secreted, the specific enzymatic pathway that processes proguanylin into its bioactive GN form has not been entirely understood yet (93). The GN peptide comprises four cysteine residues, enabling the assembly of two intramolecular disulfide bonds. The disulfide bonds in the peptide are essential for keeping the peptide in its proper shape, which is required for binding to the GC-C receptor (**Figure 6A**) (94). The GC-C receptor is additionally stimulated by the enteric bacterial ST peptides, which comprise 19 amino acids and three disulfide bonds and are homologs of GN (93, 95). The 3-disulfide structure of ST peptides significantly enhances their potency at the GC-C receptor, surpassing that of endogenous GC-C agonists (94). GN exists in two isoforms, a right-handed and a left-handed spiral form, which exhibits different biological activities and has varying affinities for binding to GC-C (95). The bioactive isomer of GN binds to the extracellular domain of GC-C, leading to the activation of the receptor’s intracellular domain. The biological relevance and mechanism of the interconversion of these isomers remain unknown (95). Research efforts have aimed to identify the cellular source of guanylin, with early studies proposing that enteroendocrine cells, such as goblet cells, paneth cells, tuft cells, and enterocytes, were responsible for producing these peptides due to their proposed hormonal function. However, discrepancies between studies and techniques have made it difficult to determine a definitive source (93, 96, 97). Although the stimuli that trigger proguanylin secretion remain poorly characterized, evidence suggests a strong association with the salt consumption (98).

**Figure 7:**
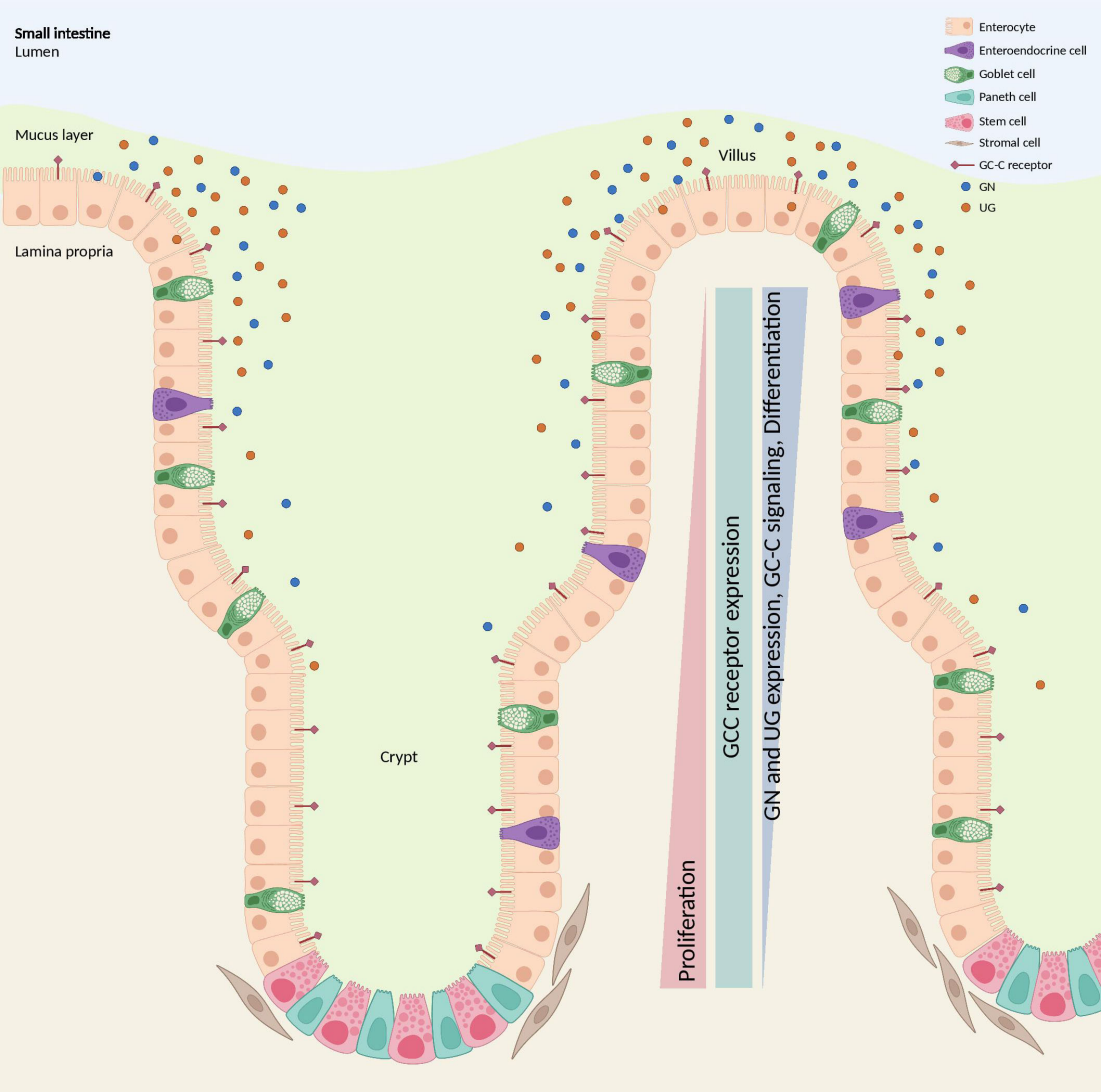
GC-C signaling regulates crypt-villus proliferation. While the GC-C receptors are expressed along the crypt-surface axes, the endogenous ligands GN and UG are produced by differentiated cells bordering the lumen and are absent in crypts. GC-C signaling opposes the proliferative gradient along that vertical axis by restricting cell growth in the crypts and promoting the maturation of differentiated cells. GC-C, guanylate cyclase c; GN, Guanylin; UG, Uroguanylin. Created with BioRender.com.

The loss of GN has been shown to have detrimental effects on the intestinal epithelial cells that produce GC-C, disrupting the homeostatic mechanisms necessary for organizing the crypt-villus axis. The GC-C expression remains consistent across the crypt-to-villus axis. However, the endogenous ligands GN and UG are secreted in an ascending gradient, with the highest concentration in the differentiated villus compartment and the lowest in the proliferating crypt compartment. This gradient restricts proliferation and reprograms metabolism in crypts, contributing to maintaining a healthy intestinal epithelium (99) (**Figure 7**). The perturbation of GN production leads to deficiencies in cellular proliferation, sensing and repair of DNA damage, and metabolic programming, collectively contributing to the development of tumors (85, 100). GN stands out as a frequently absent gene product in the context of colorectal tumorigenesis and is regarded as one of the initial occurrences in the progression of intestinal tissue transformation (101, 102). In an investigation involving a cohort of patients diagnosed with stage I-III CRC, it was observed that both *GUCA2A* mRNA and peptides exhibited a loss or substantial reduction in cancerous tissues when compared to adjacent healthy tissues in more than 85% of the cases (102). Moreover, lower circulating levels of proguanylin were observed in individuals with obesity, and levels increased following Roux-en-Y gastric bypass surgery, suggesting potential links to metabolism or food intake (103). Consistent with these results, GN expression and peptide levels were reduced in mice fed a high-fat diet. Conversely, the risk of developing colon cancer due to obesity was decreased when GN was forcibly re-expressed (100).

Bashir et al. (51) found that *GUCA2A* expression was lost in both tubular and serrated adenomas compared to their corresponding normal colon tissues. The study also suggested that this loss may silence *GUCY2C*, which could lead to the development of microsatellite instability tumors (51) (**Table 3**). Blomain et al. (38) reported a similar finding, demonstrating that the loss of GN expression occurs early in APC-dependent tumors in both humans and mice, whereas the GC-C receptor remains intact. In 2022, Liu et al. (60) conducted an integrated bioinformatics analysis to identify hub genes associated with colorectal adenocarcinoma and assess their prognostic significance. They collected colon adenocarcinoma (COAD)/rectum adenocarcinoma (READ) data from the Cancer Genome Atlas (TCGA) database (https://www.cancer.gov/ccg/access-data). Additionally, they utilized the gene expression profile of GSE25070 from the Gene Expression Omnibus (GEO) database (https://www.ncbi.nlm.nih.gov/geo/). This data collection aimed to explore differentially expressed genes between colorectal adenocarcinoma and normal tissues. Their analysis led to the identification of a *GUCA2A*-*CLCA1*-*CLCA4* gene signature that could accurately predict the prognosis of CRC patients (60). Another study by Ming Li et al. (59) utilized three microarray datasets (GSE23878, GSE33113, and GSE41328) to investigate the relationship between gene expression and patient outcomes in CRC. They identified four differentially expressed genes (DEGs), including *GUCA2A*, *ADH1C, CLCA4*, and *CXCL8*, all of which were associated with significantly lower overall survival in CRC patients (59). Chen et al. (16) analyzed 437 mutation data from colon cancer samples and discovered a positive correlation between *GUCA2A* expression and patient survival. In a separate study, Hases et al. (55) used data from TCGA database analyzed clinical samples of colorectal tumors, and matched noncancerous adjacent tissue from CRC patients to identify sex-specific biomarkers for CRC. *GUCA2A* was identified as a prognostic biomarker for CRC, specifically in males (55). Zhang et al. (52) identified *GUCA2A* as a hub gene significantly correlating with patients’ overall survival (OS). Furthermore, they conducted qPCR analysis and found that *GUCA2A* is significantly downregulated in tumor and metastatic tissues when compared to adjacent normal tissues (52).

### 3.3 GUCA2B

The human gene encoding uroguanylin (UG) termed Guanylate Cyclase Activator 2B (*GUCA2B*), is located on chromosome 1 in humans (1p34.2), which consists of 3 exons. The expression of *GUCA2B* mRNA resembled that of *GUCA2A* expression, indicating that *GUCA2A* promoter-proximal and upstream super-enhancer elements synchronize the expression of both genes (49). UG peptide comprises 16 amino acids and, equal to GN, consists of two disulfide bonds between positions seven and 15 (**Figure 6B**) (93). This peptide is produced and expressed in enterochromaffin cells predominantly in the proximal part of the small intestine (76, 104). Plasma UG circulates as both propeptide (proUG) and active forms, while plasma GN circulates mainly as proGN (105). Renal tubular brush border membrane-associated enzymes convert the inactive proUG through a proteolytic process into the bioactive UG, resulting in high amounts of UG in the urine (106). Of significance, the expression of UG is invariably lost early during neoplastic transformation in the intestine (107, 108). By the oral administration of human UG, Shailubhai et al. (22) demonstrated that the number of polyps was reduced by 50% in ApcMin/+ mice (mice carrying mutations in the APC gene) as well as the progression of polyps into adenocarcinoma was decreased. In agreement with this finding, Basu et al. (80) showed that oral administration of UG prevented ApcMin/+ mice from forming adenomas, their progression to colon tumors, and the development of inflammation-induced colonic tumors in ApcMin/+ mice. As a bioinformatics research sample, Chu et al. (109) used prediction analysis of microarray (PAM), artificial neural network (ANN), classification and regression trees (CART), and C5.0 to identify gene expression profiles of CRC and normal mucosa. They pooled 16 datasets containing 88 normal mucosal tissues and 1186 CRCs and identified the top eight differential genes in CRCs, including suppressor genes *GUCA2B*, *CA7*, *IL6R SPIB*, *CWH43*, and *AQP8*; and oncogenes *TCN1* and *SPP1* (109).

Recently, Yang et al. (67) performed a comprehensive *in silico* analysis and discovered that patients with *DNAH7* missense mutations might benefit more from ICIs (**Table 4**). Through establishing the protein-protein interaction (PPI), they identified the top key genes associated with the *DNAH7* mutation, including *GUCA2B*, *AQP8*, *MS4A12*, and *ZG16* (67). These results may shed light on the possible role of the *GUCA2B* gene as a predictor of ICIs response in CRC patients, which requires further studies in this regard. Ebadfardzadeh et al. (56) conducted a comprehensive analysis of gene expression patterns in four microarray datasets (GSE113513, GSE10950, GSE25070, and GSE37182) available in GEO, as well as miRNA expression profiles. After analyzing the data, the researchers identified a total of 43 DEGs, including ten hub genes; *GUCA2B*, *GUCA2A*, *CLCA4*, *SLC26A3*, *KRT20*, *CLCA1*, *MAOA*, *MS4A12*, *AQP8*, and *ADH1A*. Additionally, the team identified four differentially expressed miRNAs that compromise *miR-502-3p*, *miR-552*, *miR-490-5p*, and *miR-423-5p*. Based on their bioinformatics analysis, the DEGs identified in this study could serve as significant biomarkers in the molecular mechanisms of CRC development, potentially aiding in developing novel strategies for predicting, screening, and diagnosing CRC patients (56). In another study, the GSE41258 and GSE81558 microarray datasets were analyzed by Han et al. to search for specific molecular targets for diagnosis and prognosis in CRC patients (63). A total of 53 DEGs were identified between CRC and normal colorectal tissues. The list was narrowed to ten hub genes, including *GUCA2A*, *GUCA2B*, *GCG*, *SST*, *MS4A12*, *PLP1*, *CHGA*, *PYY*, *VIP*, and *CLCA4*. However, just *CLCA4* and *MS4A12* expression levels had a statistically significant effect on CRC patients’ OS (63). In the same vein, the GSE50760 and GSE104178 datasets were further mined to identify potential target genes correlated with CRC pathogenesis. There were 53 overlapped DEGs in these three datasets. The researchers utilized the String-db online tool to assess the possible interaction of the shared DEGs (110). Ultimately, they identified a significant sub-network of ten genes, including *GUCA2B*, *GUCA2A*, *GCG*, *BEST4*, *UCN3*, *SST*, *NPY*, *PYY*, *OTOP2*, and *TMEM82*. However, expression levels of *GUCA2B* and *GUCA2A* have no correlation with the OS of patients with CRC in this study either (58). Nomiri et al. (66) sought to identify a potential target for CRC therapy using Weighted Gene Co-expression Network Analysis (WGCNA) to investigate key modules, hub genes, and mRNA-miRNA regulatory networks correlated with CRC. The study found 372 genes to be considerably positively associated with CRC (r = 0.98, P-value = 9e-07) and determined 22 hub genes through survival and differential expression analyses. Among these hub genes, *GUCA2B*, *C2ORF88*, and *CCDC68* were found to play crucial roles in the overall survival rate of CRC patients. The expression analysis was consistent with RT-qPCR results, demonstrating a significant reduction in the expression of *GUCA2B* in CRC tissues. In addition, the study identified top microRNAs correlated with *GUCA2B*, and receiver operating characteristic (ROC) analyses indicated that *GUCA2B* has a high diagnostic performance for CRC (66). In 2022, Rappaport et al. (49) examined a GUCY2C ligand transcriptional silencing mechanism by β-catenin/TCF signaling. In this regard, they performed RNA sequencing analysis of conditional human colon cancer cell lines of β-catenin/TCF signaling to map the core Wnt-transcriptional program. As an important result of this investigation, a novel locus control region regulated by APC-β-catenin-TCF silences *GUCA2A* and *GUCA2B* transcription was discerned. With this discovery, they introduced a unique opportunity to reverse *GUCY2C* ligand silencing and oppose tumorigenesis in the context of mutant Wnt signaling (49). A former study by Pucci et al. (111) has established that higher *B4GALNT2* gene expression in CRC patients strongly predicts a good prognosis for their cancer outcome. In another study, to further illustrate the biological importance of this gene in CRC, Pucci and her group obtained gene expression data of 626 COAD/READ samples from the TCGA database and divided CRC patients based on *B4GALNT2* expression into two different groups, including higher and lower expressers. Patient stratification revealed that the higher expression cohort displayed a concomitantly high level of other genes associated with a favorable prognosis, such as *GUCA2B, ZG16, ITLN1*, and *BEST2* (64).

## 4 Discussion and future prospects

Early detection of colorectal cancer is crucial, as it enables access to a broader range of therapeutic interventions and significantly impacts patient survival. Our review study highlights the critical involvement of the guanylate cyclase-c signaling pathway in the early stages of colon carcinogenesis. Investigating the signaling pathway expression in individuals with high-risk colon polyps or early stages of colon carcinoma may be beneficial. This could assess the GC-C axis as a diagnostic marker in a cohort study.

To our knowledge, no studies have yet measured the levels of guanylin and uroguanylin in the serum of patients with CRC. Further investigation is needed to fill this gap by examining the serum levels of these proteins in diverse patient cohorts, as well as their correlation with the cancer stage. This information could provide valuable insights into the severity of CRC and help predict disease progression.

In light of the promising advancements in immunotherapy for cancer treatment, investigating the immune roles of *GUCA2A*, *GUCA2B*, and *GUCY2C* genes could provide valuable insights into their potential as targets for immunotherapy. Specifically, *GUCY2C* has been found to play a regulatory role in intestinal inflammation and inflammatory bowel disease pathology. At the same time, *GUCA2A* has been associated with the immune signature of CRC. Therefore, further investigations into the immune-related functions of these genes could pave the way for developing more effective immunotherapies for treating CRC.

Recent studies have suggested that targeting *GUCA2A*, *GUCA2B*, and *GUCY2C* genes could be a promising approach for treating CRC. For instance, linaclotide (a GC-C receptor agonist) has been used in previous studies for the prevention of CRC (112), and is currently being evaluated in phase I (NCT01950403) and phase II (NCT03796884) clinical trials for the treatment of CRC. Further research into the use of drugs targeting the GC-C receptor or those that affect these genes could lead to developing more effective and targeted treatments for CRC patients. Many studies have examined the effects of GC-C receptor agonists in treating CRC. However, there is a shortage of drugs specifically targeting the expression of *GUCA2A* and *GUCA2B* genes related to CRC. Although research on GN and UG has mainly been done through bioinformatics, it is crucial to carry out *in vivo*, *in vitro*, and human studies on *GUCA2A* and *GUCA2B* to better comprehend how these genes contribute to CRC growth and advancement.

Finally, the future prospects for the Guanylate cyclase-C signaling pathway in oncology are hopeful, with potential applications in the diagnosis, prognosis, and treatment of CRC. Further research and clinical trials are demanded to fully realize these peptides’ potential and develop novel drug delivery systems that enhance their therapeutic efficacy.

Although research studies have unveiled the significance of the GC-C signaling pathway in colorectal cancer, its practical application in clinical oncology remains constrained. To address this limitation, we propose some future directions for integrating this pathway into clinical settings. Achieving a comprehensive understanding of the GC-C signaling pathway in gastrointestinal cancer, particularly colon cancer, necessitates a comprehensive evaluation of all its constituent components (GUCY2C, GUCA2A, and GUCA2B). Recognizing the transformative impact of systems biology approaches in elucidating complex biological phenomena, we advocate for a multi-layered assessment encompassing this pathway’s transcriptomics, proteomics, metabolomics, and metagenomics, employing high-resolution methods.

## 5 Conclusion

To sum up, this systematic review provides a comprehensive summary of the current state of knowledge on the potential role of the guanylyl cyclase-c receptor (GC-C) and its endogenous ligands, Guanylin and Uroguanylin, in the development and progression of colorectal cancer (CRC). Clinical studies have shown that GC-C expression and its activators are associated with patient outcomes, suggesting their potential as prognostic tools. The review also underscores the therapeutic potential of GC-C targeted therapies in CRC treatment. The findings suggest that combining GC-C targeted therapies with other treatment modalities could enhance their effectiveness and overcome drug resistance. Hopefully, these results will serve as a valuable resource for researchers and clinicians developing new and effective therapies for CRC patients, ultimately leading to improved patient outcomes. Further research is warranted to determine the efficacy and safety of GC-C targeted therapies, and the reviewed literature provides a foundation for future studies in this field.

## Data availability statement

The original contributions presented in the study are included in the article/supplementary material. Further inquiries can be directed to the corresponding author.

## Author contributions

MoeinP: Methodology, Writing – original draft, Writing – review & editing. AA: Methodology, Writing – original draft. PJ: Writing – original draft. AS: Writing – original draft. MobinP: Writing – original draft. YT: Writing – original draft. ZS: Conceptualization, Supervision, Writing – review & editing.
